# Assessment of the content and quality of YouTube videos on retinopathy of prematurity: a cross-sectional study

**DOI:** 10.5935/0004-2749.2024-0214

**Published:** 2024-12-26

**Authors:** Ceren Durmaz Engin, Dilhan Karaca, Taylan Ozturk

**Affiliations:** 1 Department of Ophthalmology, Democracy University Buca Seyfi Demirsoy Education and Research Hospital, Izmir, Turkey; 2 Department of Ophthalmology, Karadeniz Eregli State Hospital, Zonguldak, Turkey; 3 Department of Ophthalmology, Tinaztepe University Hospital, Izmir, Turkey

**Keywords:** Retinopathy of prematurity, YouTube, Information dissemination/methods, Online education, Internet access, Social media/instrumentation, Information seeking behavior, Internet/statistics & numerical data, Consumer health information, Social networking, Reproducibility of results

## Abstract

**Purpose:**

This study aimed to evaluate the quality and reliability of YouTube videos as an educational resource about retinopathy of prematurity.

**Methods:**

Videos were sourced from YouTube using the search terms “retinopathy of prematurity” and “premature retinopathy” with the default settings. Each video was assessed on the following metrics: views, likes, dislikes, comments, upload source, country of origin, view ratio, like ratio, and video power index. The quality and reliability of the videos were evaluated by two independent researchers using the DISCERN questionnaire, the JAMA benchmarks, the Global Quality Score scale, the Health on the Net Code of Conduct, and the Ensuring Quality Information for Patients scale.

**Results:**

The study assessed 92 videos, the majority of which (42 videos, 45.7%) originated from the United States. Most of the videos focused on screening, pathophysiology, and diagnosis of retinopathy of prematurity (61.9%). The primary contributors were medical organizations (19 videos, 20.6%), nonacademic health channels (19 videos, 20.6%), and physicians (15 videos, 16.3%). Significant differences were found between the DISCERN (p=0.003), JAMA (p=0.001), Global Quality Score (p=0.003), Health on the Net Code of Conduct (p=0.006), and Ensuring Quality Information for Patients (p=0.001) scores among different video sources. However, the key video metrics did not differ. Using the DISCERN and Global Quality Score scales, the overall YouTube video content on retinopathy of prematurity was rated as moderate in quality. Using the Health On the Net Code of Conduct and Ensuring Quality Information for Patients scales, it was rated as high quality. Strong correlations were observed between the scores on all of the scales (p<0.001).

**Conclusion:**

Videos from medical organizations and healthcare centers were of a higher quality than those from nonmedical sources. Despite the varied foci of each evaluation scale, the strong correlation between them indicates that they provide reliable and comprehensive assessments of the quality of informational content.

## INTRODUCTION

Retinopathy of prematurity (ROP) is an eye disorder that affects premature infants. It can cause lifelong visual impairment and is a leading cause of childhood blindness^(^1^)^. A full understanding of ROP is crucial for health professionals and parents, who are key members of the ROP care team. Educating parents and helping them to comprehend the diagnosis is essential. Despite their crucial role, parents are rarely present during ROP procedures. This can lead to anxiety and a need for information. In busy clinical settings, healthcare providers may struggle to address all parental queries, prompting parents to seek information from other sources.

The internet has become a critical resource for those seeking information on health issues, with many people conducting online research before seeking medical advice. A survey indicated that 72% of internet users utilize the web to gather health information^(^2^)^. Bianco et al. found that 84.7% of parents research their children’s medical conditions online. Lee et al. identified a significant shift in recent years toward the internet as the initial source of health information^(^3^,^4^)^. A preference for visual content over text-based information has been demonstrated, explaining the popularity of YouTube as a source of health information^(^5^)^. As the leading videosharing site, YouTube hosts videos with medical content from a wide array of sources, including patients, physicians, other healthcare professionals, medical information sites, and various organizations^(^6^)^. However, the lack of peer review for YouTube videos raises concerns about their reliability and quality, particularly conditions such as ROP, which can lead to blindness if treatment is delayed. To date, only a few studies have investigated the utility and quality of ROP video content, and these studies have used a limited number of scales^(^7^,^8^,^9^)^. Therefore, this study aims to assess YouTube videos on ROP using five standardized, validated scales to identify any improvements needed in the online dissemination of accurate and reliable ROP information.

## METHODS

### Screening and selection of videos

For this cross-sectional study, we performed YouTube searches on August 20, 2023, using the search terms “retinopathy of prematurity” and “premature retinopathy”. We kept the site’s default search settings, which were set to organize videos by relevance. The initial 100 videos returned for each search term were assessed for inclusion in the study. Our exclusion criteria were duplicates of videos already included videos that were commercial in nature, those in languages other than English, those without narration, those irrelevant to ROP, and videos shorter than 15 seconds. The search process was conducted without signing into a user account and with a cleared search history. This study was approved by the local ethics committee.

To facilitate effective statistical analysis, the videos were grouped into three source categories: videos by healthcare centers, physicians, and public and private hospitals; videos from medical organizations such as the American Academy of Ophthalmology and the All India Ophthalmological Society; and videos from nonmedical sources, including nonacademic channels, pharmaceutical companies, and patient-created content. The key parameters recorded for each video were view count, video duration, number of likes, number of dislikes, number of comments, time since upload, country of origin, and educational value. Videos offering scientifically valid information on any aspect of ROP, including its etiology and pathogenesis, screening and diagnosis, treatment, and prognosis, were classed as useful. Those presenting unverified scientific information were classed as misleading, and videos with incorrect data were classed as harmful.

Popularity assessment metrics included the view ratio, the like ratio, and the video power index (VPI). The view ratio was derived by dividing the total number of views by the time between the video’s upload and the analysis date. The like ratio was calculated using the formula [(likes × 100)/(likes + dislikes)]. VPI, a metric designed to gauge each video’s impact, was calculated as (likes/(likes + dislikes)) × view count. These metrics provided insights into viewer engagement and the overall influence of each video on the platform.

### Assessment scales

Two researchers (CDE and D.K.) independently evaluated each video, focusing on their content, quality, and reliability. Both researchers are ophthalmologists, and they have 10 and 5 years of experience in the diagnosis and treatment of ROP, respectively. The assessment tools used were the DISCERN instrument, the Journal of the American Medical Association (JAMA) benchmarks, the Ensuring Quality Information for Patients (EQIP) tool, the Health On the Net Code of Conduct (HONCode), and the Global Quality Score (GQS).

The DISCERN instrument, created by Charnock et al., assesses the quality of health information for reliability and treatment content^(^10^)^. It consists of 16 questions, with a possible score of 1 to 5 on each. These evaluate reliability and treatment details, with the final question assessing overall quality without a designated score. The total possible score ranges from 16 to 75. Based on the score, the video quality is then categorized as excellent (63-75), good (51-62), moderate (39-50), poor (27-38), or very poor (16-26).

The JAMA benchmark evaluates the credibility of online health resources across four factors: authorship, attribution, disclosure, and currency. Each factor is scored 0 or 1, with a maximum score of 4^(^11^)^. Higher scores indicate greater quality.

The GQS uses a five-point Likert scale to assess the source’s quality, clarity, and information flow^(^12^)^. Scores range from 1 (poor quality) to 5 (excellent quality). Based on the score, the video quality is then categorized as high (4 or 5), medium (3), or low (1 or 2).

The HONCode, established by the Health on the Net Foundation in 1998, is a set of standards for trustworthy online health information. It assesses information sources on eight principles: authoritativeness, complementarity, privacy, attribution, justifiability, transparency, financial disclosure, and advertising policy^(^13^)^. Each principle is rated from 0 to 2, with a maximum score of 16.

The EQIP tool was designed by health professionals and patient information managers to evaluate the quality of health information on websites and in patient leaflets. It is a 20-item scale that assesses the source for accuracy, balance, structure, design, and readability^(^14^)^. Question responses are binary, with responses of yes or no given for each. One point is given for each yes response and zero points for each no response, with a total possible score of 20.

The assessors were blinded to each other’s scores to maintain objectivity. To ascertain the reliability and consistency of the scores given by the two assessors, intraclass correlation coefficients (ICCs) were calculated. The scores of the two assessors on each scale were averaged for statistical analysis purposes.

### Statistical analysis

The statistical analysis in this study was conducted using SPSS Statistics for Windows, version 25 (IBM Corp., Armonk, NY, USA). The descriptive statistics used to summarize the data were counts and percentages for categorical data and means and standard deviations for quantitative data. The normality of data distribution for each variable was determined using the Shapiro-Wilk test. Normally distributed continuous variables were analyzed by one-way analysis of variance. Non-normally distributed variables were analyzed using the KruskalWallis test. Post-hoc analyses were adjusted with Bonferroni correction. Categorical variables were analyzed using chi-square or Fisher’s exact tests, as appropriate. To assess inter-rater reliability, ICCs were calculated using a two-way mixed-effects model. The level of statistical significance was p<0.05.

## RESULTS

**Figure 1:**
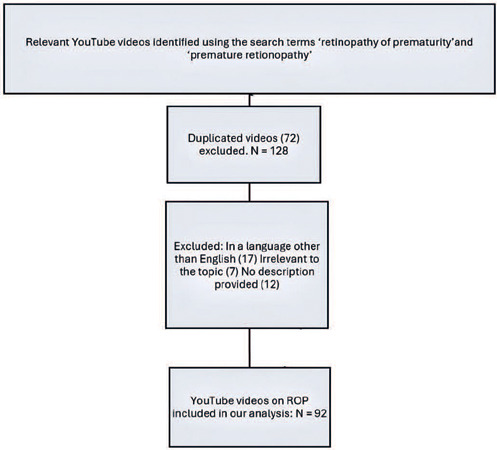
Schematic representation of the methodology for screening and selecting retinopathy of prematurity-related video content on YouTube.

After applying our exclusion criteria, 92 of the initial 200 videos retrieved. A flow chart illustrating the video selection process is provided in figure 1. The median view count of the sample of videos was 2607.5 (interquartile range [IQR]: 502.5-8845.0), with an median length of 7.01 minutes (3.11-16.15) and a view ratio of 1.63 (0.51-7.80). The like ratio was 100 (97.35-100), and the median number of likes was 25.5 (5.25-77.75). The median VPI was 2.08 (0.53-8.21).

Assessment of the sample’s distribution across countries of origin showed that 42 (45.7%) of the 92 videos were from the United States, and 36 (39.1%) were from India. The other countries of origin (n=14, 15.2%) were the United Kingdom, Canada, and the Philippines, among others.

Medical organizations were responsible for uploading 19 videos (20.6%), nonacademic channels for 19 videos (20.6%), physicians for 15 videos (16.3%), public or private hospitals for 25 videos (27.1%), pharmaceutical companies for five videos (5.4%), and patients for nine videos (9.7%). For a more robust analysis, videos uploaded by physicians and hospitals were combined into a healthcare centers category, medical organizations were kept as a separate category, and the remaining videos were combined into a nonmedical sources category. In terms of content, 37 videos (40.2%) were primarily intended for medical education, 26 (28.3%) were designed for patient education, 20 (21.7%) were suitable for the education of both patients and medical and healthcare students and practitioners, and nine (9.7%) provided content focused on patients’ experiences. Regarding their utility, 79 videos (85.9%) were classed as useful, 12 (13.0%) as misleading, and just one (1.1%) as harmful. In most of the videos (n=56, 60.9%), the person directly informing the viewer or consulted by the presenter was an ophthalmologist. In 12 (13.0%) videos, this person was a neonatologist.

**Figure 2:**
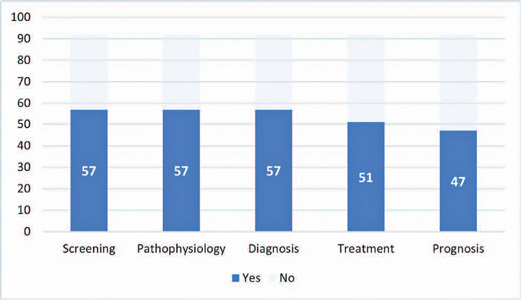
Quantitative distribution of videos across various theme categories.

In 57 (61.9%) of the videos, the focus was the screening process for ROP, a further 57 (61.9%) focused on pathophysiology, and 57 (61.9%) on diagnosis. The distribution of focal topics is illustrated in figure 2.

The scores obtained by the two observers showed were similar. The ICC values for the DISCERN, JAMA, GQS, HONCode, and EQIP scales were 0.858, 0.911, 0.908, 0.833, and 0.911, respectively. These all indicate strong inter-rater reliability. The mean DISCERN score for the whole sample of videos was 39.99 ± 16.25 (range: 15-72), the mean JAMA score was 2.15 ± 0.91 (1-4), the mean GQS was 2.84 ± 0.91 (1-4), the mean HONCode score was 7.49 ± 3.41 (1-15), and the mean EQIP score was 59.19 ± 15.05 (16.60-87.50).

The mean scores for the DISCERN, JAMA, GQS, HONCode, and EQIP scales, categorized by video source, are presented in table 1. Our comparisons of scales across the three source groups found significant between-group differences in the mean DISCERN (p=0.003), JAMA (p=0.001), GQS (p=0.003), HONCode (p=0.006), and EQIP (p=0.001) scores. Post-hoc analysis indicated that the differences in DISCERN (p=0.002), JAMA (p=0.001), and HONCode (p=0.004) scores primarily stemmed from a disparity in video quality scores between videos uploaded by medical organizations and those by nonmedical sources. The differences in the GQS were between both videos uploaded by medical organizations and nonmedical sources (p=0.007) and between those uploaded by healthcare centers and nonmedical sources (p=0.018). Similarly, EQIP scores differed between videos from medical organizations and nonmedical sources (p=0.001) and between those from healthcare centers and nonmedical sources (p=0.021). The only statistically significant difference in video metrics between the three video source groups was in the number of comments (p=0.049). The assessment metrics for videos in each video source category are presented in table 1.

**Table 1:** Comparison of scores for information quality on the DISCERN, JAMA, GQS, HONCode, and EQIP scales of YouTube videos on retinopathy of prematurity by video source

	Healthcare Centers (n=40)	Medical Organizations (n=19)	Nonmedical Sources (n=33)	p-value
**Scale** ^a^				
**DISCERN score**	40.25 ± 14.58	49.58 ±19.07	34.15 ± 14.06	**0.003**
**JAMA score**	2.23 ± 0.73	2.68 ± 1.05	1.76 ± 0.86	**0.001**
**GQS**	3.00 ± 0.90	3.21 ± 0.78	2.42 ± 0.86	**0.003**
**HONCode**	7.60 ± 2.97	9.37 ± 3.63	6.27 ± 3.35	**0.006**
**EQIP score**	61.26 ± 13.81	67.16 ± 12.59	52.11 ± 15.10	**0.001**
**Characteristics** ^b^				
**View count (n)**	3437.00 (657.25–11172.25)	4844.00 (869.00–9699.00)	1886.00 (323.50–5738.50)	0.116
**Length (min)**	6.48 (3.24–18.66)	8.42 (3.31–68.50)	7.010 (2.14–10.89)	0.250
**Age (d)**	1349.50 (908.25–2401.00)	1190.00 (709.00– 2952.00)	1223.00 (560.00–2358.00)	0.506
**Likes (n)**	37.50 (9.00–91.00)	27.00 (4.00–78.00)	13.00 (2.00–66.50)	0.125
**Dislikes, (n)**	0 (0–3.00)	0 (0–4.00)	0 (0–2.50)	0.869
**Comments, (n)**	5.50 (0–14.75)	0 (0–3.00)	1.00 (0–9.00)	**0.049**
**View ratio**	2.12 (0.51–10.50)	2.68 (0.63–10.04)	1.03 (0.31–6.63)	0.357
**Like ratio**	100.00 (98.02–100.00)	100.00 (95.68–100.00)	100.00 (97.2–100.00)	0.697
**Video power index**	2.08 (0.50–9.35)	2.67 (0.63–10.27)	1.16 (0.37–6.62)	0.722

^a^= ANOVA for normally distributed variables, mean ± SD; ^b^= Kruskal-Wallis test for non-normally distributed variables, median (IQR)ANOVA, analysis of variance; EQIP= Ensuring Quality Information for Patients; GQS= Global Quality Score; HONCode= Health On the Net code; IQR= interquartile range; JAMA= Journal of the American Medical Association; SD= standard deviation; VPI, video power index.

There was a strong positive correlation between DISCERN scores and other quality metrics, including JAMA scores (r=0.760, p<0.01), HONCode (r=0.816, p<0.01) scores, and EQIP scores (r=0.792, p<0.01), indicating consistent evaluation across these tools. There were no significant correlations between the scores on any of the scales and video metrics, except for a significant weak positive correlation between like ratios and EQIP scores (p<0.05). There was a moderate negative correlation between like ratio and view ratio, while both showed a moderate positive correlation with VPI (p<0.001 for both). The VPI showed a near-perfect correlation with the view ratio (r=0.999, p<0.01), highlighting the strong relationship between views and the overall strength of video engagement. The results of our analysis of the correlations between the assessment scales and the key metrics are summarized in table 2.

**Table 2:** Correlations between information quality scores on the DISCERN, JAMA, GQS, HONCode, and EQIP scales and video metrics for YouTube videos on retinopathy of prematurity

	DISCERN score	JAMA score	GQS	HONCode score	EQIP score	View ratio	Like ratio	VPI
**DISCERN score**	1.00							
**JAMA score**	0.760**	1.00						
**GQS**	0.836**	0.731**	1.00					
**HONCode score**	0.816**	0.861**	0.780**	1.00				
**EQIP score**	0.792**	0.793**	0.804**	0.863**	1.00			
**View ratio**	0.015	-0.034	-0.030	-0.046	-0.066	1.00		
**Like ratio**	0.208	0.134	0.240	0.145	0.228*	-0.597**	1.00	
**VPI**	0.010	-0.046	-0.055	-0.058	-0.088	0.999**	-0.572**	1.00

*Correlation significant at the p<0.05 level (two-tailed).

**Correlation significant at the p<0.01 level (two-tailed).

EQIP= Ensuring Quality Information for Patients; GQS= Global Quality Score; HONCode= Health On the Net Code; JAMA= Journal of the American Medical Association; VPI= video power index.

## DISCUSSION

YouTube is frequently used as a source of information due to its easy accessibility and open access. However, because it is an open platform, there is no system in place to ensure the reliability and quality of content. Any registered user can upload videos without meeting pre-established content criteria. In this study, we aim to inform clinicians about the content and quality of YouTube videos related to ROP, utilizing reliable and well-validated quality assessment tools such as the HONCode and EQIP scales to ensure a comprehensive analysis.

To date, three studies have evaluated YouTube videos on ROP^(^7^,^8^,^9^)^. The earliest of these, by Sahin et al., primarily assessed the sources of videos and their utility and did not use specific assessment scales. They found 64% of the videos useful, with healthcare professionals as the source of 70% of those^(^7^)^. Conversely, our findings suggest that 85% of the videos are useful information sources for both medical practitioners and patients, with just one video identified as harmful. Unlike Sahin’s study, which found physicians (31%) and hospitals (25%) to be the primary sources of videos, Uzun et al. found universities/ non-profit organizations (36.9%) and physicians (31.5%) to be the top contributors^(^8^)^. Our analysis aligns with the latter, identifying healthcare centers as the primary source of ROP-related videos. The increase in online webinars and virtual meetings by healthcare centers and medical organizations during the COVID-19 pandemic, along with improved content, may account for the differences between the sources found by Sahin et al. and those found by Uzen et al. and us. The most recent of the three previous studies was by Raffa et al. This evaluated ROP-related YouTube videos in Arabic and found 72.5% useful. However, the videos mainly targeted medical professionals^(^9^)^. In contrast, we found that, while 40% of the videos in our sample were specifically for medical professionals, the remainder were sufficiently comprehensible to also benefit patients. This difference may stem from the greater number of ROP videos in English than in Arabic and the tendency for more frequently viewed videos to rank more prominently in search results, which likely increases the visibility of patient-friendly content in English due to its broader and more diverse viewership.

Our study revealed that most YouTube ROP videos presented in English originate from the United States (42 videos, 45.7%) and India (36 videos, 39.1%), despite the increasing prevalence of ROP in regions like Latin America, Eastern Europe, and the Middle East. This may be because the use of English search terms biases the returned results towards videos from English-speaking countries. Additionally, local search algorithms favor English-language content. The extensive use of social media by US healthcare entities likely influenced this pattern further^(^15^,^16^)^. India’s notable representation might be due to its widespread use of English and significant ROP prevalence^(^17^)^.

While the majority of the videos assessed addressed screening, pathophysiology, and diagnosis, a slightly smaller proportion covered treatment, and even fewer addressed prognosis. This is perhaps because screening, pathophysiology, and diagnosis are broader topics that can be more easily discussed by medical and nonmedical sources. Additionally, from a clinical perspective, not all physicians involved in ROP screening and diagnosis are involved in its treatment. Sahin et al. observed that videos uploaded by surgeons or practitioners contained more detailed information about treatment procedures. In contrast, those posted by free clinics usually provided more basic information on ROP^(^7^)^. Similarly, Çakmak et al. evaluated YouTube videos on pancreatic cancer and found that 36% of the videos provided general information, 6% included diagnostic information, and just 2% addressed treatment^(^18^)^. Another YouTube content analysis study on neonatal sepsis revealed that, while most provided general information about the disease, only a small proportion (14%) of the videos discussed treatment^(^19^)^. The relative paucity of content on treatment modalities, disease course, and post-treatment prognosis may be attributable to the fact that knowledge of these subjects is more exclusive to qualified physicians or healthcare institutions.

We observed that videos from non-medical sources had lower view and like ratios and lower VPIs than those from medical sources, although the differences were not statistically significant. Previous studies suggest that, while physician-produced videos are more reliable, they tend to attract fewer views, likely because patients find them harder to understand^(^5^,^20^)^. However, with the increased use of videos by medical professionals for educational purposes, the discrepancies between the videos uploaded by medical and nonmedical sources may reduce over time.

The mean DISCERN score in our study was categorized as moderate. We observed a significant difference in the DISCERN scores of videos from the three source groups, with nonmedical sources having significantly lower scores. There was no significant difference between the DISCERN scores of videos from healthcare centers and medical organizations. In contrast to our findings, Uzun et al. and Raffa et al. found no significant difference between videos from medical and nonmedical sources or between those by physicians and nonphysicians^(^8^,^9^)^. This variation may stem from the different subgroups compared in these studies. Analyses of YouTube videos on diverse medical conditions have generally shown that nonmedical sources have lower DISCERN scores. This could be due to the emphasis on treatment information in the DISCERN scale, a topic best addressed by medical professionals^(^21^,^22^,^23^,^24^)^. The lack of standardized video quality categories for JAMA score ranges has led to variations in previous studies, with mean scores of 0.74 ± 0.82 labeled as lowest, 1.3 ± 0.4 as poor, and 1.65 ± 0.89 as fair. The maximum possible score on the JAMA scale is 4. As the videos in our sample had a mean JAMA score of 2.15 ± 0.91, we classified the video quality as good. Our results roughly align with those of previous research, although several studies found no difference in the GQS of videos from medical and nonmedical sources^(^16^,^21^,^25^)^.

As with JAMA, there are no standardized criteria for video quality assessment using the HONCode and EQIP scales. Parmar et al. rated the quality of Ahmed glaucoma valve surgical videos as excellent, with a mean HONCode score of 6.86 ± 0.75^(^26^)^. Vought et al. assigned a moderate quality rating to their mean EQIP score of 15.1 for refractive surgery videos. However, this was markedly lower than the EQIP score of 53.01 reported by Kim et al. for videos on temporomandibular disorders^(^27^,^28^)^. Based on our assessments, we conclude that, overall, the videos assessed in this study are of high quality when evaluated with the EQIP and HONCode scales. These two scales have been used more widely in the last few years. Despite the lack of standardized criteria for video assessment, they have been found to contribute to a higher scientific quality of studies, having a higher number of questions than other scales, which provides a more detailed evaluation of content for accuracy, balance, structure, readability, privacy, and transparency^(^27^,^29^)^.

The DISCERN, JAMA, GQS, HONCode, and EQIP scores showed a strong positive correlation with each other (p≤0.001). This is in accord with the findings of several other studies^(^16^,^30^)^. Interestingly, no correlation was found between quality assessment scores and video metrics, except for a weak positive correlation between like ratios and EQIP scores (Table 2). Given these findings, future content creators should ensure that high-quality content is presented in an engaging manner accessible to the general population.

The strengths of our study include its comprehensive approach to video quality evaluation using five distinct scales, its focus on the latest content, and its analysis of English-language videos, which are the most widely accessed globally. This contrasts with previous studies that have evaluated only video metrics, treatment-specific content, or videos in languages other than English. This study thereby offers a broader and more nuanced understanding of the quality of YouTube videos on ROP^(^7^,^8^,^9^)^. While the DISCERN, JAMA, and GQS questionnaires are commonly used in quality assessments, the use of HONCode and EQIP in previous research has been limited^(^22^,^24^,^25^)^. We found significant differences in the scores on both scales between nonmedical video sources and the two medical video source groups.

This study had several limitations. First, we must acknowledge the subjective nature of video evaluation. However, the high ICC value indicates strong inter-rater agreement, strengthening the reliability of our results. This minimization of subjectivity can be attributed to the clarity and design of the scales used. Second, we did not assess the audio or visual quality of the videos. In instances where this is poor, it is likely to have significantly affected our viewer engagement metrics. Third, the study can be regarded only as a snapshot of YouTube content at a single point in time, as the platform’s content is subject to change. This challenges the permanence of the study’s findings. Changes to the site may affect the results obtained using our search terms over time. Additionally, our analysis used standard search settings, reflecting the content most accessible to the average user.

In conclusion, our study suggests that while ROP-related YouTube videos are generally of satisfactory quality, it is crucial for parents to consult medical professionals for individualized information. YouTube can be an accessible and useful medical resource, especially when videos are produced by medical professionals. However, the information that videos provide should be comprehensible and useful to the target viewer, addressing the specific needs of healthcare professionals and patients separately.
